# Familial melanoma: genetic, clinical, and dermoscopic insights and follow-up

**DOI:** 10.3389/fmed.2026.1906952

**Published:** 2026-07-27

**Authors:** Alise Emma Raika, Maria Elena de las Heras Alonso, Diana Sproge, Elga Bataraga

**Affiliations:** 1 Department of Dermatology and Venereology, Riga Stradins University, Riga, Latvia; 2 University Hospital Ramón y Cajal, Madrid, Spain; 3 Department of Medicine, University of Alcalá, Madrid, Spain

**Keywords:** BAP1, CDKN2A, dermoscopy, familial melanoma, multiple primary melanomas

## Abstract

Familial melanoma accounts for approximately 5–10% of all melanoma cases. It is characterized by an earlier age of onset. Multiple primary melanomas occur in up to a fifth of patients with a positive family history, compared to 5% in sporadic cases. Pathologically, familial melanomas tend to present at thinner Breslow depths and show a higher proportion of *in situ* lesions compared to their sporadic counterparts, with a predilection for the lower limbs. The genetic landscape of familial melanoma is heterogeneous. CDKN2A and CDK4 mutations act through UV-dependent and cell cycle disruption pathways, while BAP1, POT1, and TERT mutations are considered UV-independent. Beyond cutaneous melanoma, germline mutations confer an increased risk for additional malignancies, including pancreatic cancer, renal cell carcinoma, mesothelioma, and neural system tumors, necessitating multidisciplinary surveillance and genetic counseling for affected families. Dermoscopically, CDKN2A mutation carriers predominantly present with superficial spreading melanoma, displaying atypical pigment networks, irregular dots and globules, regression structures, and a blue-white veil. Co-occurrence of MC1R variants may produce more subtle, structureless presentations with a lower total dermoscopy score, posing additional diagnostic challenges. Sequential digital dermoscopy and total body photography remain the cornerstones of surveillance, with follow-up every 3–6 months recommended for mutation carriers. All patients should receive structured education on photoprotection, including the use of broad-spectrum SPF50+ sunscreen and sun avoidance measures.

## 1 Introduction

The first documented case of familial melanoma was reported in 1820 by William Norris, who described a 59-year-old man with melanoma, a high total body nevus count, and a family history of melanoma (1). More than 100 years later, Lynch and Krush (2) made a pivotal contribution by documenting an association between pancreatic cancer, multiple nevi, and melanoma within families, and in 1978, the term “Familial Atypical Multiple Mole Melanoma” (FAMMM) was proposed (3). In 1994, CDKN2A (cyclin-dependent kinase inhibitor 2A) was identified as the first major familial melanoma susceptibility gene, marking a pivotal era in the understanding of familial melanoma (4).

Currently, familial melanoma is defined according to regional melanoma incidence. It has been reported that approximately 5–10% of melanoma cases are associated with a positive family history (5–9). Of these familial cases, approximately 20% are attributable to high-risk, high-penetrance germline mutations (5, 10).

Familial melanoma classification may be adjusted according to regional (geographical) melanoma incidence, with the diagnostic threshold increasing proportionally to the background population risk (6, 11, 53).

- *Rule of 2 (low–moderate incidence regions, e.g., Southern Europe)*: ≥2 primary melanomas in a single individual and/or ≥1 invasive melanoma with ≥1 additional diagnosis of melanoma or pancreatic cancer in first- or second-degree relatives on the same familial side.
- *Rule of 3 (moderate–high incidence regions, e.g., Northern Europe and North America)*: ≥3 primary melanomas in a single individual and/or ≥1 invasive melanoma with ≥2 additional related diagnoses in first- or second-degree relatives.
- *Rule of 4 (very high incidence regions, e.g., Australia and New Zealand)*: ≥4 primary melanomas in a single individual and/or ≥1 invasive melanoma with ≥3 additional related diagnoses in first- or second-degree relatives.

These criteria reflect the higher background melanoma incidence in regions with greater cumulative UV exposure. Notably, the presence of multiple primary melanomas alone may warrant genetic testing, regardless of the regional incidence or family history (6, 7, 11, 12). It should be noted that some regions (particularly across Asia and Africa) remain underrepresented in the current literature, which may affect the generalizability of these thresholds.

Some patients with familial melanoma clinically present with multiple cutaneous melanomas at an early age (7–9). Recognition of familial melanoma extends beyond its cutaneous manifestations, as many hereditary melanoma syndromes are associated with an increased risk for other malignancies. This multisystem cancer risk warrants comprehensive surveillance strategies and multidisciplinary management approaches for affected patients and their families (11, 13).

This review aims to provide a comprehensive overview of familial melanoma, encompassing its historical recognition, genetic foundations, clinical manifestations, and dermoscopic characteristics. We emphasize practical insights for clinicians regarding patient identification, genetic testing indications, and management strategies for individuals at hereditary risk of melanoma.

## 2 Genetic background

### 2.1 High-penetrance genes

#### 2.1.1 CDKN2A

Cyclin-dependent kinase inhibitor 2A (CDKN2A) is considered the most clinically significant gene, accounting for approximately 20–40% of all familial melanoma cases. Therefore, CDKN2A is the most widely studied gene associated with familial melanoma and inherited melanoma susceptibility (10, 11, 14).

CDKN2A encodes two distinct tumor suppressor proteins: p16 (INK4A; RB pathway) and p14 (ARF; p53 pathway), both of which play crucial roles in cell cycle regulation, causing uncontrolled cell cycle entry and impaired apoptosis and DNA damage response (7). Accordingly, CDKN2A mutations confer a dramatically increased risk of melanoma.

Research by Cust et al. (15) reported an 87-fold increased risk of melanoma among CDKN2A mutation carriers compared with the general population in the United Kingdom and a 31-fold increased risk in Australia. The penetrance of CDKN2A mutations is quite notable, reaching approximately 30% by age 50 and 67% by age 80. CDKN2A mutation carriers typically develop melanoma at a younger age, with an average age at diagnosis of 30–40 years (6). In comparison, the average age at diagnosis in the general population is over 50 years (16).

Key clinical features associated with CDKN2A mutations include multiple primary melanomas, early-onset melanoma, and an increased risk of pancreatic cancer (11).

In addition, CDKN2A mutation carriers have a substantially higher risk of developing multiple melanomas than the general population (17).

#### 2.1.2 CDK4

Cyclin-dependent kinase 4 (CDK4) mutations are much rarer and have been identified in fewer than 20 families reported in the literature to date. Pathogenic CDK4 mutations disrupt the interaction between CDK4 and p16INK4a (5). The clinical phenotype of cutaneous melanoma seen in families with CDK4 variants is similar to that seen in families carrying a CDKN2A variant—namely, the presence of multiple dysplastic nevi and an elevated risk of developing multiple primary melanomas (11, 17).

#### 2.1.3 BAP1

BAP1 mutations are associated with an increased risk of uveal melanoma, cutaneous melanoma, malignant mesothelioma, and renal cell carcinoma, and specific forms of benign skin lesions (including BAPomas). An estimated 63% of patients with germline BAP1 variants develop at least one of these cancers before the age of 53. Although important in the context of cutaneous melanoma, BAP1 mutations are particularly important in uveal melanoma (5, 18, 19).

#### 2.1.4 Telomere maintenance genes

Several genes involved in telomere maintenance have emerged as important familial melanoma susceptibility genes. Among these, POT1 mutations primarily affect the protein’s DNA-binding domain and increase the risk of multiple melanomas, particularly between the ages of 25 and 80 (5, 20).

TERT promoter mutations are rare (found in approximately 1% of familial melanomas), but highly penetrant and associated with clinically aggressive early-onset melanomas occurring between the ages of 18 and 46 (5, 21).

ACD and TERF2IP mutations increase the risk of multiple primary melanomas, typically diagnosed at a young age (during the second decade of life) (5).

### 2.2 Intermediate-penetrance genes

#### 2.2.1 MC1R

MC1R variants are among the most common genetic factors associated with melanoma susceptibility. MC1R is involved in the regulation of skin and hair color in humans.

R alleles of MC1R are associated with a 28% increased risk of developing melanoma, independent of other phenotypic factors. The risk increases with the number of variants—doubling in subjects with a single variant and reaching sixfold higher levels in those with two or more variants (5, 22).

Importantly, MC1R variants double the risk of melanoma in CDKN2A mutation carriers, with the risk being threefold higher in carriers of red hair color (RHC) variants (23).

#### 2.2.2 MITF

The MITF p.E318K variant confers up to a fivefold increased risk of developing melanoma and is present in 1% of individuals of European descent. This mutation is associated with high nevus counts, early-onset melanoma before the age of 40, and non-blue eye color. Carriers also have an increased risk of renal cell carcinoma (5).

## 3 Clinical characteristics of familial melanoma

### 3.1 Early age of onset

One of the most striking features of familial melanoma is the earlier age of diagnosis. The average age of diagnosis of melanoma in CDKN2A-mutated patients is between 30 and 40 years, whereas the average age of diagnosis in the general population is approximately 50 years (5, 24).

Screening for melanoma in high-risk families should commence at age 10, with baseline total body skin examinations, including dermoscopic evaluation of atypical lesions (11, 14).

### 3.2 Multiple primary melanomas (MPMs)

Multiple primary melanomas (MPMs) tend to develop sporadically in approximately 5% of patients who have had a previous melanoma, compared with 19% in patients who have a family history of melanoma. The risk of developing a second primary melanoma for patients with a single melanoma is estimated to be higher, and 40% of multiple primary melanomas occur synchronously (5, 25).

The pattern of MPM development varies. Some patients present with synchronous melanomas at initial diagnosis, while others develop malignant lesions months or decades after the initial diagnosis. A family history of melanoma combined with MPMs creates a multiplicative increase in the probability of carrying a germline mutation (26).

### 3.3 Melanoma characteristics

Familial melanomas display distinct pathological features. Melanomas belonging to the sporadic melanoma group had a significantly higher Breslow thickness, indicating that familial cases tend to be diagnosed at earlier, thinner stages. There were also significantly higher numbers of *in situ* melanomas in the familial group than in the sporadic group (17, 27).

Considering melanoma sites, a significantly higher percentage of familial melanomas are found on the lower limbs (17).

Melanomas may arise within pre-existing nevi or develop *de novo* on previously normal-appearing skin. Even in patients with numerous atypical nevi, the majority of melanomas arise de novo (26).

In familial melanoma, the number of melanocytic nevi in affected individuals can vary widely. Some family members present with a high nevus burden, while others have comparatively few, despite the shared genetic risk. Genetic susceptibility to nevus formation contributes to familial melanoma, which is characterized by high nevus density and reflects a polygenic risk for nevogenesis (28).

However, familial melanoma can also arise from the inheritance of high-penetrance pathogenic variants (such as CDKN2A) that increase melanoma risk regardless of the total nevus count. Individuals with few nevi can still carry a strong genetic predisposition to melanoma (29). Studies of melanoma families have shown that CDKN2A mutation carriers also tend to have increased counts of atypical nevi, although the relationship with total nevus number is not consistent across all nevus types, highlighting further phenotypic heterogeneity (10, 30).

Regarding the importance of UV damage, in some familial melanomas, the combination of genetic inheritance and UV-related risks plays an important role. The most obvious UV-dependent variants involve CDKN2A, CKD4, and MC1R mutations. The latter is associated with a classic patient phenotype: red hair, fair skin, freckling, and an increased melanoma risk connected to UV-dependent DNA damage, due to a lack of eumelanin (6, 22, 31). Germline BAP1, POT1, and TERT mutations are considered to be UV-independent (5, 18, 20, 32).

### 3.4 Associated malignancies

The association between pancreatic cancer and melanoma is often observed in CDKN2A-mutated melanoma families. Studies have reported up to a 40-fold increased risk of pancreatic cancer in patients with CDKN2A mutations compared with the general population (11, 24, 33). As this gene mutation is most frequently found to be altered in familial pancreatic cancer (10), this association has led to the recognition of the melanoma–pancreatic cancer syndrome as a distinct clinical entity (29). In addition to pancreatic cancer, neural system tumors, non-small cell lung cancer, breast cancer, and leukemia are also linked to CDKN2A mutations (10). BAP1 mutations are found in patients with renal carcinoma, mesothelioma, cholangiocarcinoma, lung cancer, and other carcinomas. POT1 mutations are associated with thyroid cancer and a Li-Fraumeni-like syndrome (10, 29) (see Table 1).

**Table 1:** Familial melanoma susceptibility genes, clinical characteristics, and associated malignancies.

Penetrance	Gene	Clinical characteristics	Other tumor associations
High-penetrance genes	CDKN2A	Multiple primary melanomas; early onset; atypical nevi may be increased.	Pancreatic cancer; melanoma–pancreatic cancer syndrome; neural system tumors; NSCLC; breast cancer; leukemia.
	CDK4	Rare; CDKN2A-like phenotype; dysplastic nevi; multiple primary melanomas; often superficial spreading melanoma.	Non-melanoma skin cancers; breast, pancreas, ovarian, cervical, and stomach cancers; FAMMM.
	BAP1	Cutaneous/uveal melanoma; BAPomas; at least one associated cancer before age 53.	Uveal melanoma; malignant mesothelioma; RCC; bladder carcinoma; lung carcinomas; breast and thyroid tumors; neuroendocrine tumors; paraganglioma; BCC; rhabdoid meningiomas; cholangiocarcinoma.
Telomere maintenance genes	POT1	Multiple melanoma phenotype; broad age range: 25–80.	Angiosarcoma; glioma; CLL; thyroid and colorectal cancers; Li-Fraumeni-like syndrome.
	TERT	Rare; early-onset aggressive melanoma, reported at age 18–46 years.	Myeloproliferative neoplasms; bladder, colon, prostate, testis, breast, kidney, and CNS tumors.
	ACD/TERF2IP	Young-onset multiple primary melanomas.	B-cell lymphoma; leukemia; esophagus, breast, lung, kidney, cervix, ovary, colon, bladder, and prostate cancers.
Intermediate-penetrance genes	MC1R	R/RHC variants independently increase melanoma risk and further amplify risk in CDKN2A carriers.	Non-melanoma skin cancers.
	MITF p.E318K	~1% in Europeans; high nevus count; melanoma before age 40; non-blue eye color.	Renal cell carcinoma; pheochromocytoma; paraganglioma reported.

Adapted from the previously compiled references. In-table citations were removed for readability. Retained reference numbers: (5, 10, 11, 13, 16–24, 26–31, 33–40, 43, 44, 47–52).

BCC, basal cell carcinoma; CLL, chronic lymphocytic leukemia; FAMMM, familial atypical multiple mole melanoma; NSCLC, non-small cell lung cancer; RCC, renal cell carcinoma; RHC, red hair color.

## 4 Dermoscopic patterns in familial melanoma

Melanomas arising in familial melanoma patients demonstrate dermoscopic features consistent with their histopathologic subtypes. Patients with germline CDKN2A mutations have significantly more superficial spreading melanomas than nodular or lentigo maligna melanomas.

The predominance of superficial spreading melanoma in CDKN2A carriers typically manifests with classic melanoma dermoscopic criteria:

- Atypical pigment network.
- Irregular dots and globules (varying in shape, size, and distribution).
- Blue–white veil.
- Irregular streaks (pseudopods, radial streaming).
- Regression structures (*peppering*, white scar-like areas).
- Amelanotic variants (with polymorphous vascular pattern, milky-red areas, etc.) (7, 25, 34).

However, melanomas in CDKN2A carriers with red hair color MC1R variants may present with structureless areas and can be more difficult to diagnose, even with comparative dermoscopic approaches (31, 35). MC1R carriers present with a lower total dermoscopic score (TDS), compared to non-carriers who present with an atypical pigment network (35).

Nevus-associated melanomas, which may be more common in familial settings given the high nevus burden, can demonstrate distinctive dermoscopic patterns. These include multicomponent patterns with areas of benign-appearing nevus alongside regions showing melanoma-specific features, multifocal hyperpigmentation, or peripheral melanoma patterns surrounding a central benign-appearing nevus (36, 37).

In BAP1 carriers, melanomas may arise as amelanotic or hypomelanotic lesions that demonstrate dermoscopic features including polymorphous vascular patterns, white structureless zones, linear looped vessels, linear irregular vessels, milky-red areas, and shiny white structures. In these variants, the absence of melanin pigments renders pigment-based criteria uninformative (38, 39).

In patients with MITF mutations, Ciccarse et al. have analyzed three dermoscopic patterns in melanomas: non-specific, globular-homogeneous, and reticular-homogeneous. MITF positive patients showed more non-specific patterns compared to MITF negative patients (40) (see Table 2).

**Table 2:** Dermoscopic features in familial melanoma.

Gene	Key dermoscopic features	UV association
CDKN2A standard	Atypical pigment network Irregular dots and globules Blue-white veil Irregular streaks (pseudopods, radial streaming) Regression structures (peppering, white scar-like areas) Amelanotic variants (polymorphous vessels, milky-red areas)	Partial
MC1R	Structureless areas predominate Reduced pigmentation and fewer dermoscopic structures Subtle atypical vessels Lower total dermoscopy score (TDS) Atypical network absent or subtle	Yes
CDK4	Similar to CDKN2A (atypical network, regression) Multiple atypical nevi background	Partial
BAP1	Amelanotic/hypomelanotic lesions Polymorphous vascular patterns White structureless zones Linear looped and irregular vessels Milky-red areas Shiny white structures	No
MITF	Non-specific pattern (most common in MITF-positive patients) Globular-homogeneous pattern Reticular-homogeneous pattern	No
Nevus-associated	Multicomponent pattern Benign nevus areas alongside melanoma-specific features Multifocal hyperpigmentation Peripheral melanoma pattern surrounding central benign nevus	Not specified

## 5 Follow-up for high-risk patients

Familial melanoma patients, particularly those with multiple nevi or known pathogenic genetic mutations, require especially attentive early melanoma monitoring (29, 41, 42). It should also be taken into consideration that, because familial melanoma predisposition genes are associated with a broader spectrum of malignancies, affected individuals and their relatives should be offered genetic counseling and multi-organ surveillance (6, 43).

In monitoring melanomas, standard diagnostic equipment used in clinical practice—digital dermoscopy along with total skin photography—has been shown to aid the detection of melanoma in these high-risk patients (42). Patient education regarding daily UV protection and routine self-examinations is also an essential component in the management of familial melanoma risk groups (44).

There are several factors that indicate the increased risk of cutaneous melanoma in patients and the necessity for digital monitoring. The most prominent factor associated with the highest relative risk is the presence of more than 60 melanocytic nevi in a patient, as the relative risk of melanoma increases with the number of common nevi. A factor of considerable importance is the presence of an identified CDKN2A mutation or other high-risk melanoma-associated genetic variants. CDKN2A carriers should be monitored regardless of their nevi count. Early melanomas in these patients particularly benefit from sequential digital dermoscopy monitoring for early detection (31, 35).

Patients with more than 40 nevi were also considered eligible for monitoring, but only when other risk factors are present (personal history of melanoma, red hair and/or an identified MC1R mutation, or a history of organ transplantation) (41).

Regarding dysplastic nevi and familial melanoma, the evidence does not show a single critical number of dysplastic nevi present to be clinically significant; however, any dysplastic nevi increase the risk of melanoma in these individuals (6, 11, 14, 41).

Follow-up intervals are not strictly standardized; however, the most widely adopted schedule for high-risk patients is surveillance every 6 months with digital dermoscopy and total skin photography, with some protocols extending to 12 months. Whereas for patients with germline mutations or multiple primary melanomas, closer follow-up every 3–6 months is recommended (42, 45–47).

Despite the molecular diversity of UV-related and UV-independent oncogenic pathways, all familial melanoma patients should be educated on sun protection practices. Patients should be advised to avoid sunbathing and tanning, including the use of sunbeds/tanning beds, and to use appropriate UV protection. Patients should apply a broad-spectrum SPF 50 sunscreen 20 min before sun exposure in an adequate amount and reapply sunscreen every 2 h and after swimming or sweating. Avoiding sun exposure during peak solar hours, seeking shade, using clothing (preferably with UPF protection), wide-brimmed hats, and sunglasses is also recommended (48, 49) (see Table 3).

**Table 3:** Indications for genetic testing in suspicion of familial melanoma.

Clinical criteria	Indication for testing
Multiple primary melanomas	≥2 primary melanomas in low incidence regions ≥3 primary melanomas in moderate incidence regions ≥4 primary melanomas in high incidence regions
Family history criteria	≥1 invasive melanoma + >1 relative with melanoma or pancreatic cancer (same familial side) ≥2 first-degree relatives with melanoma ≥1 first-degree relative with early-onset melanoma (<45 years)
Associated malignancies	Personal or family history of pancreatic cancer (up to second-degree relatives) Personal or family history of uveal melanoma Personal or family history of mesothelioma Personal or family history of renal cell carcinoma
Phenotypic high-risk features	≥60 atypical (dysplastic nevi) or ≥40 atypical nevi in the presence of BAP-1 inactivated melanocytic tumors (BAPomas)
Region-independent criteria	Any patients with multiple primary melanomas, irrespective of family history or geographic region

Testing recommended for CDKN2A, CDK4, BAP1, and MIFT/POT/ATM/TERT to be considered.

All patients referred to genetic testing should undergo pre- and post-test genetic counseling.

## 6 Conclusion

Familial melanoma accounts for 5–10% of all melanoma cases and presents with distinctive clinical features, including early onset, multiple primary melanomas, and a high number of atypical nevi, as well as an elevated risk of associated malignancies.

Since the identification of CDKN2A in 1994, our knowledge of familial melanoma susceptibility has expanded to include high-penetrance (including CDK4, BAP1, and telomere maintenance genes) and intermediate-penetrance genes (such as MC1R and MITF). However, approximately 50% of melanoma families remain genetically unexplained, suggesting that additional susceptibility genes remain to be discovered.

The future of familial melanoma diagnosis and treatment is strongly connected to risk stratification strategies that incorporate clinical phenotypes, imaging data, and genetic factors. Furthermore, advances in artificial intelligence could also be beneficial and play a valuable role in the individualized care of patients.
